# Apple Antioxidant Properties as an Effect of N Dose and Rate—Mycorrhization Involvement: A Long-Term Study

**DOI:** 10.3390/antiox11122446

**Published:** 2022-12-12

**Authors:** Barbara Łata, Sylwia Żakowska-Biemans, Dariusz Wrona

**Affiliations:** 1 Section of Basic Sciences in Horticulture, Institute of Horticultural Sciences, Warsaw University of Life Sciences—SGGW, Nowoursynowska 159, 02-776 Warsaw, Poland; 2 Department of Food Market Research and Consumption, Institute of Human Nutrition Sciences, Warsaw University of Life Sciences—SGGW, Nowoursynowska 159c, 02-776 Warsaw, Poland; 3 Department of Pomology and Horticulture Economics, Institute of Horticultural Sciences, Warsaw University of Life Sciences—SGGW, Nowoursynowska 159, 02-776 Warsaw, Poland

**Keywords:** *Malus domestica* Borkh., N nutrition, bacterial-fungal inoculum, antioxidant, HPLC, catalase

## Abstract

The genetic and/or the agronomic approaches are two main ways to enhance concentrations of biologically active compounds in fruits and vegetables. In this study, the apple antioxidant status was evaluated from the second to the fourth year after planting in relation to an increasing N-dose applied—with or without plant microbial inoculation in the field conditions. Cultivar ‘Šampion Arno’ was selected to test these relationships. In the growing season, N treatment and inoculation effects were monitored for the apple peel total phenolics and selected individual phenolic compounds ((+)-catechin, (−)-epicatechin, chlorogenic and caffeic acids, rutin and phloridzin) and total ascorbate concentration. Additionally, as an environmental stress marker measurement of glutathione reductase, ascorbate peroxidase and catalase activity were conducted. The year effect was most pronounced, while the N or applied inoculum effects were much weaker. Great differences in antioxidative enzyme activity and phenolic concentrations between years were revealed. Nitrogen fertilization reduced the fruit’s global phenolic accumulation compared to the control, but the N-effect varied depending on individual phenolic compounds, N dose and N application method. None of the tested factors influenced the ascorbate concentration. There was a certain tendency to increase antioxidant properties in the control group (without mineral N fertilization) but with the application of bio-fertilizer, which may seem promising for future research in this scope.

## 1. Introduction

The genetic and/or the agronomic approaches are two main ways to enhance concentrations of biologically active compounds in plant derived foods [1]. Genetically modified plants are poorly accepted by consumers, therefore special attention is paid to what extent agrotechnical approaches can change or enhance the content of pro-health compounds [2,3,4].

Nitrogen (N) is an essential nutrient needed for plant growth and development and one of the main nutrient related factors limiting crops’ yield and quality [5,6,7,8]. Adjusting controllable environmental factors, such as nutrient availability has the potential to shift the plant’s metabolism towards an increased production of health-promoting phytocompounds [4,6,9,10]. Soil’s N level can alter the production of secondary metabolites, however, both N excess and N deficiency are disadvantageous as they can trigger oxidative stress [11,12]. Determining the optimal N dose is one of the most difficult challenges in plant nutrition. The key aspects in relation to N fertilizers usage are low N use efficiency (NUE) and high risk to the environment as a result of volatilization or leaching [13,14]. An improvement in the NUE and mitigation of the adverse effects of N on the environment, including climate change, is one of the most important prerequisites for sustainable crop management in line with the European Green Deal strategy [7,15]. Therefore, several attempts have been made to improve the NUE in crop management system [16]. Among them, the use of bio-fertilizers is considered as a promising solution [7,15,17,18]. Bio-fertilizer is a material that is the carrier of microorganisms, living or dormant, that inhabit the plant rhizosphere or are present inside the plants [19]. Bio-fertilizers are classified based on the microorganisms present in them (bacteria, fungi) or their function. Due to the function, can distinguish N fixer, P, K solubilizers, S oxidizer, siderophore producers, organic matter decomposers and others microorganism [15,19,20]. With respect to plant nutrition, the effect of microorganism based products can be of a direct nature, when they increase a given nutrient concentration/bioavailability and their uptake by plants (e.g., N fixer), whereas indirect action relies on improving relationships between nutrients in the soil. Bio-fertilizers can influence plant supply with K and S that in turn improves N efficiency use. The introduction of bio-fertilizers into agro-technical approaches can improve soil quality and resistance, plant growth under stresses and contribute to growth resilience of the agro-ecosystem to unfavorable effects of climate change. As recently revealed, using inoculum of the specialized microorganisms, generally referred to as arbuscular mycorrhizal fungi (AMF) or plant growth promoting rhizobacteria (PGPR), can improve the overall plant performance [13,21,22,23]. Thanks to AMF, water and essential nutrients from soil are more effectively exploited by host plants since AMF helps to overcome three major factors limiting nutrient uptake, i.e., its concentration (available for plant form) at the absorbing root surface, the total surface area of roots available for uptake and the distribution of roots in the soil [24,25]. In turn, the plant nutritional status determines the resistance to stress and the quality of food after harvest and in storage [6,26]. Moreover, beneficial microorganisms can be added to the soil separately or in combination with mineral fertilizes [27,28,29,30], although the use of chemical fertilizers may have different effects depending on the physical and chemical properties of the soil, dose and fertilizer types [31,32,33]. In apple orchards, the combination of microbial biofertilizer and mineral fertilizers applied at a low rate ensured yield comparable to that obtained with a high-rate mineral fertilizer application.

Apart from influencing the plant nutritional status, it was found that AMF and PGPR applied alone or in combination can influence plant biochemistry and physiology [20,26,34]. In relation to the above, the up-regulation of several components of antioxidant machinery, such as superoxide dismutase (SOD), catalase (CAT), ascorbate peroxidase (APX), glutathione reductase (GR), ascorbic acid, glutathione, carotenoids or phenolic compounds, is considered as an interrelationships between plant and microorganism [26,35,36]. The stimulation of the antioxidant apparatus may vary depending on tissue type [34]. The involvement of AMF/PGPR in keeping the level of reactive oxygen species under control to help maintain membrane integrity and cellular stability, protects the photosynthetic apparatus, improves plant nutrient and water uptake and finally shapes yield and its quality [20,22,30].

Similarly as bio-fertilizers, mineral fertilizers, depending on the dose, form or time of application, may affect the metabolism of antioxidants [4,6,37]. The antioxidant enzymes activity is described as markers of stresses of various origins, including these regarding plant mineral nutrition [38]. SOD, CAT, GR or APX activity were frequently included testing antioxidant metabolism in abiotic stress conditions [34,35,39]. Up- or down-regulation in enzymatic antioxidants may occur due to too low or luxurious nitrogen fertilization [4,38]. Bai et al. [6] conducted meta-analysis regarding the effect of mineral nutrition on Rosaceae species’ with respect to yielding, fruit quality and some aspects of physiology/biochemistry of plant mineral nutrition. The authors concluded that N supply mainly affects fruit yield, anthocyanin synthesis and chlorophyll degradation in the fruit.

Apples are one of the world’s most important fruit crops and one of the most studied species. This is because a wide range of varieties (early and late varieties) differing in storage time, color, shape, sensory properties, antioxidant and health-promoting potential and their culinary use that can satisfy the requirements of various consumers [6]. Internal apple quality regarding several factors was widely discussed and described in detail by many authors [3,39,40,41,42,43]. However, the relationship between agronomic practices, such as fertilizers/biofertilizers management or cultivation system, and phytochemicals are less frequently studied [3,34,44]. N fertilization is often associated with a decrease in the content of biologically active compounds [4,8,45], while some studies indicate that this negative relationship can be mitigated in combination with biofertilizers [27,46]. In combination with other benefits of bio-fertilizer, using such solutions in fertilizer management can reduce the use of agrochemicals in orchards and, therefore, is worth considering. According to the best of our knowledge, there are no studies where the effect of various dosage and way of plant N nutrition in combination with the use of an effective microorganisms on the apple pro-health compounds contents were tested. Orchards are specific farms since they are long-term plantations and various research results obtained for annuals cannot be directly related to them.

Therefore, the main aim of this study was to evaluate the N effect with or without the use of biofertilizer as bacterial-fungal inoculum on the level of selected antioxidants in apples of the ‘Šampion Arno’ cultivar. The research was conducted in three consecutive growing seasons in a fully fruiting orchard.

## 2. Materials and Methods

### 2.1. Experimental Design, Weather and Soil Conditions

**Figure 1:**
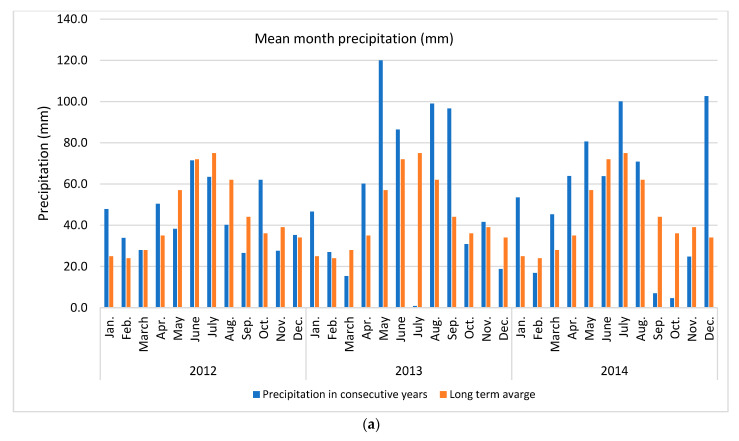

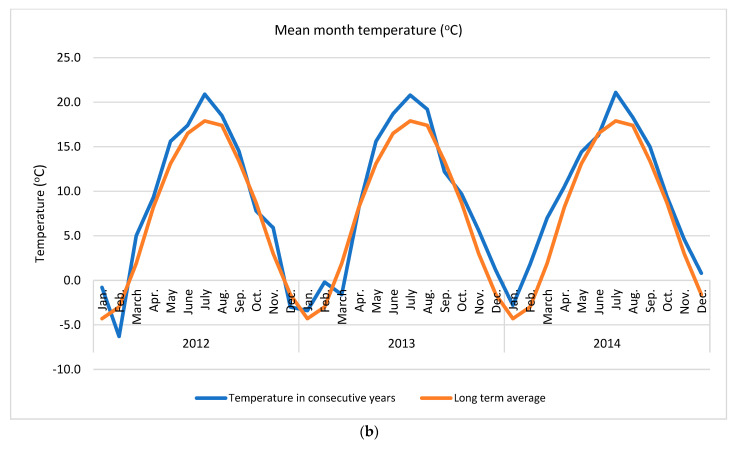
Weather conditions accompanying the study (**a**) mean month precipitation and (**b**) mean month temperature. Long term averages were calculated for the period 1982–2012.

Apple of ‘Šampion Arno’ cultivar was collected from the experimental orchard in Warsaw at Wilanów in Central Poland (52°9′36.1″ N + 21°5′58.2″ E) in three growing seasons (2012, 2013 and 2014). The temperature and rainfall data in subsequent years was collected using the Vantage Pro 7 weather station (Davis Instruments, Hayward, CA, USA) (Figure 1).

The plants of cultivar ‘Šampion Arno’ (*Malus domestica* Borkh.) were planted in 2011 on M.9 rootstock on a loamy alluvial soil (2.5% humus) within a split-block experimental design. The trees were planted in two rows, one of them consisted of non-inoculated trees (N-INO, non-inoculated trees) and the second inoculated trees (INO) with AMF (mycorrhizal arbuscular fungi) and PGPR (plant growth-promoting rhizobacteria).

Biofertilizer Micosat F (CCS Aosta, Quart, Italy) at a total concentration of 106 CFU g^−1^ in a powder form was used in the study. The microorganisms’ composition in the bio-fertilizer was as follows: AMF: *Glomus mosseae* GP11, *G. intraradices* GB67, *G. viscosum* GC41, PGPR: *Bacillus subtilis* BA41 and *Streptomyces* spp. SB19. Bio-fertilizer at 10 g·tree^−1^ dose was applied to a depth of 30 cm during tree planting. The procedure was repeated every year, namely a 2 g m^−2^ dose of the inoculum was applied three times during growing season at 21 day intervals, starting at the beginning of May. In addition to biofertilizer and increasing N doses with different way of N application were also tested, namely: N0: control, without N fertilization; N1: 50 kg N·ha^−1^, only herbicide strip was fertilized; N2: 50 kg N·ha^−1^, the entire plot surface was fertilized; N3: 100 kg N·ha^−1^, the entire surface of the plot was fertilized; N4, in total 100 kg N·ha^−1^ was applied, but divided into two doses: the first dose of 50 kg N·ha^−1^ was applied to the entire surface of the plot in early spring and the second one 50 kg N·ha^−1^ at the end of May.

The presented research is part of a long-term and multifaceted study regarding apple trees fertilization. Therefore, in the floor management system, the more details into experimental design and data on mycorrhizal parameters were recently presented [21]. In brief, the values of mycorrhizal frequency, absolute mycorrhizal intensity and relative mycorrhizal intensity were higher every period the roots were tested [21].

### 2.2. Fruit Sample Collection and Analysis

The apples were harvested in 2012 on September 26 and in 2013 and 2014 on September 19. Fruits of similar size were picked from the outer layer, avoiding the tops and bottom of the trees, from four trees per repetition (in total 16 fruits from one plot), a total repetition of three for each treatment (30 plots under study).

Due to the a high correlation coefficient between apple peel and whole fruit antioxidant concentrations, the epidermic zone of the apple fruit was collected according to procedure recently described [41].

The collected apple peels were immediately frozen in liquid nitrogen and stored at −80 °C. Fruit tissue was homogenized in liquid nitrogen into fine powder and the tissue powder was used for antioxidant extraction.

### 2.3. Enzymatic and Non-Enzymatic Antioxidant Measurements

Activity of glutathione reductase (GR), ascorbate peroxidase (APX) and catalase (CAT) was measured spectrophotometrically (HITACHI UV-Vis spectrophotometer, model U2900, Kyoto, Japan, purchased from Dynamica Sci. Ltd., Milton Keys, UK).

The tissue powder was weighed (200 mg) and suspended in 100 mmol potassium phosphate buffer (5 mL, pH 7.8) containing Triton X–100 (0.5%), insoluble polyvinylpolypyrrolidone (PVPP) and ascorbate (5 mmol). The mixture was centrifuged at 48,000× *g* for 20 min at 4 °C. Enzyme activity (GR: EC 1.6.4.2, absorption coefficient 6.2 mmol^−1^ cm^−1^; APX: EC. 1.11.1.11, absorption coefficient 2.8 mmol^−1^ cm^−1^; CAT: EC 1.11.1.6, absorption coefficient 39.4 mmol^−1^ cm^−1^) was determined in a total volume of 1 mL by monitoring the decrease in absorbance at 240, 290 and 340 nm for CAT, APX and GR, respectively [40]. Enzyme activity was calculated using the appropriate absorption coefficient and expressed in nanokatals per gram dry weight (nkat g^−1^ DW).

Total phenolics content (TPC) was determined using Fast Blue BB (4-benzoylamino-2,5-dimethoxybenzenediazonium chloride hemi [zinc chloride] salt, FBBB) [47]. Tested tissue was extracted twice in a methanol solution in ultrasonic bath for 30 min at room temperature [48]. Extracts or standards (with a volume of 1000 µL) were transferred to spectrophotometric cells, then FBBB (100 µL 0.1% FBBB) and NaOH (100 µL 5% NaOH) were added. After one hour incubation, absorption of obtained solutions was measured at 420 nm. The TPC was expressed in gallic acid equivalents (GAE) per kg^−1^ DW.

Identification and quantification of individual phenolic compounds were conducted with an HPLC system from Waters Company (System Breeze, Milford, MA, USA), which consisted of binary pump (type 1525), Waters In-Line Degasser AF, an autosampler with a thermostat scale of 4–40 °C (M 717 PLUS), UV-VIS detector (M 2487) and a 5–85 °C thermostat for the column (Peltier). Phenolic compounds were separated on a Symmetry C18 column (250 mm × 4.6 mm with 5 μm packing, Waters Co., Taunton, Ireland) protected with a corresponding guard column (Symmetry C18, 5 μm, 3.9 mm × 20 mm) by applying an aqueous solution of 0.01 molar phosphoric acid (solvent A) and 100% methanol (solvent B). The column was maintained at 25 °C. The sample injection volume was 20 μL. The flow rate and time of one separation were 1 mL min^−1^ and 35 min, respectively. Phenolics were detected at 280 nm [48]. Quantification was based on an external standard calibration curve based on commercially available chlorogenic acid (1,3,4,5-tetrahydroxycyclohexanecarboxylic acid), (+)-catechin, (−)-epicatechin, rutin (quercetin-3-rutinoside hydrate) (Sigma-Aldrich Chemie, Steinheim, Germany) and phloridzin dihydrate (Fluca, Steinheim, Germany).

The total concentration of ascorbate (L-AA + DHAA; L-ascorbic and dehydroascorbic acids, respectively) was measured after complete oxidation of L-AA to DHAA with ascorbate oxidase [41] using HPLC (Waters Co.) technique. DHAA was derivatized with o-phenylenediamine and the reaction product was detected as a fluorescent compound at 450 nm by excitation at 350 nm. Separation was conducted using Symmetry C18 column (250 mm × 4.6 mm, 5 mm, Waters Co., Milford, MA, USA) coupled with a fluorescent detector (Waters 2487) under isocratic conditions. The mobile phase contained 20% methanol and 800 mmol K_2_HPO_4_ with a pH of 7.8. The flow rate was 1 mL min^−1^. The concentrations of ascorbate were calculated using standard curve.

### 2.4. Statistical Analysis and Presentation of Data

The data obtained were analyzed using a multifactor analysis of variance (ANOVA) within the Statistica 13 software package (StatSoft, Cracow, Poland). The significance of differences between the means was tested using Tukey’s or LSD tests.

## 3. Results and Discussion

### 3.1. Weather Conditions and Growing Season Effect on Apple Peel Antioxidants

In presented study various N dosage with the use of an effective microorganisms (PGPR + AMF) on the apple pro-health compound concentrations in three subsequent growing seasons were tested. It was found that the strongest effect was year effect compared to other factors, such as various N dose and application way, with or without biofertilizer (Table 1). The distribution of precipitation (a) and temperature (b), the two important factors for plant growth, in the research years in relation to the long-term average (years 1982–2012) are presented in Figure 1. The sum of temperatures in March–September (plant growing season for cultivar tested) and the average monthly temperature in 2012 and 2014 were about 16% higher (an increase of 2 °C) compared to the long-term average. The sum of precipitation in 2012 was close to the long-term average, while in 2014 it was higher by about 19%. At the same time, in 2012 the distribution of precipitation was more favorable than in 2014. In the case of rainfall, not only was the precipitation amount a key issue, but also its distribution during the growing season and the intensity in conjunction with the soil type. Soil type can influence antioxidant metabolism [49]. High rainfall in heavy loamy alluvial soil, as in case in this study, may deteriorate the air conditions and further conditions for the uptake and of nutrient transport. An increase in temperatures and droughts are two main indicators of climate change which simultaneously strongly affect plant growth conditions [7,22]. These conditions are strictly related to the uptake of essential nutrients, which in turn affect overall plant performance [50]. Unfavorable external surroundings generate oxidative stress, which plants react to by activating various antioxidant systems [12]. It is a complex process in which antioxidant enzymes alone or together with non-enzymatic antioxidants maintain the balance between the generation and removal of reactive oxygen species [11].

In this study, the activity of two enzymes, GR and APX, participating in the ascorbate-glutathione cycle during the H_2_O_2_ removal and maintaining the active, redox state [11] of these compounds was the highest in 2014, followed by 2012 and 2013. The differences in enzyme activity between the years were statistically significant. Ascorbate concentration was only slightly increased in 2012 and 2014 compared to 2013. However, unlike the GR and APX activity, CAT activity was the highest in 2012 and the lowest in 2014. A study on a great number of apple cultivars revealed a considerably higher variation in enzymatic compared to non-enzymatic antioxidants between subsequent growing seasons with a special emphasis on GR activity [40]. Increased antioxidant enzyme activity seems to be related to the acclimatization process to plant habitat conditions regarding maintenance of non-enzymatic antioxidants in an appropriate redox state and concentration. However, the efficiency of this process may depend on the duration of unfavorable conditions.

Similar to the antioxidant enzymes activity, the concentration of phenolic compounds was strongly influenced by the growing season (‘year effect’, Table 1). Unfavorable environmental conditions, especially those related to temperature and water, are a stimulus that activates plant genetic, biochemical or physiological responses that mitigate the effects of the stress factors. An increase the content of secondary metabolites, such as phenolics, and/or an increase in tissue enzymatic activity was recorded under water limitation and drought stress in several studies [51,52,53].

The phenolic accumulations might be related to their photoprotective function under conditions of high light and UV irradiation [43]. Mignard et al. [54] stated that solar radiation had positive effect on apple flesh ascorbic acid, total phenolic and flavonoid contents.

In turn, Li et al. [55] demonstrated that solar radiation influenced ascorbic acid only in the apple peel, while Bui et al. [56] indicated that a higher accumulation of phenolics and ascorbate took place in the sun-exposed part of peel as compared to the shaded part. Significant differences in apple antioxidant content were obtained in five-year study and the weather conditions was indicated as the most significant factor [57].

In presented study the phenolic concentrations were significantly higher in 2012–2013 compared to 2014. In 2013, the average temperature in September was significantly lower both in relation to the long-term average and in relation to 2012 and 2014. It is difficult to indicate the factor that influenced the phenolic accumulation in 2012. The temperature influences the color of apples, especially the red-skinned cultivars. Anthocyanins responsible for the coloration are one of the main subgroups of polyphenols. Hot dry summers do not favor fruit color development [9]. Unfortunately, the global pool of anthocyanins has not been specified in this study, which would help explain some aspects of the year effect.

Due to the fact that the weather and soil conditions consist of a number of components that interact with each other, both a comprehensive assessment and the view of individual components in terms of their impact on plant biochemistry/physiology is difficult. A multivariate model fitted with the several apple accessions and climate features during five consecutive years (2014–2018) was established by Spanish researchers [54]. They found that bioactive compound values tended to decrease in general with a higher temperature, while they increased with precipitation and solar radiation. On the other hand, maintaining higher concentrations of antioxidants can be an effective strategy for plants to mitigate the harmful effects of ROS [38]. Taking into account other long term studies on the influence of various factors regarding the antioxidant metabolism, simultaneously ignoring the tissue type which is the most impacting factor [41], the year (soil and weather variables) frequently override other factors, including agro-technical approaches [3,4,40,41,57]. However, the type, strength and duration of the environmental stress factor can finally determine the change direction i.e., up- or down- regulation of antioxidant machinery.

### 3.2. Apple Peel Antioxidant Status as a Function of N Nutrition and Bacterial-Fungal Inoculum

In our studies, the range of nitrogen nutrition was from 0 to 50 up to 100 kg ha^−1^, including various application way. In the examined N range, fertilization did not result in unequivocal changes in the activity of antioxidant enzymes (Table 2).

Compared to the control (N0), the GR and APX activity increased significantly at the dose of 100 kg N ha^−1^ (N3) and it decreased when the dose was split into two (N4) and applied on two dates. Variation in GR and APX activity between N3 and N4 treatments were, however, only a trend. In the N4 treatment, the activity of the GR is similar to the GR activity of the control group. In turn, in case of N4 treatment and the APX activity it is still considerably higher compared to control. As a rule, more efficient nitrogen use occurs with split doses. A one-time application means greater nitrogen losses and a higher concentration of salt in the soil just after fertilizer sowing what could be harmful for plants [58].

Whereas there were no significant differences between N0, N1 and N2 treatments in GR and APX activity. The CAT activity in N treatments was close to control. Significant changes in the antioxidant enzyme activity along with better N supply were reported in kiwiberry fruits [4]. The author concluded that N-dependent fluctuations of GR and APX may affect the relatively stable level of ascorbate or glutathione as these enzymes are involved in their regeneration. This mechanism of action may explain the stable concentration of ascorbate irrespective of soil N fertility in this study (Table 2). There were also lack of seasonal variability in the total ascorbate content combined with significant fluctuations in the activity of antioxidant enzymes (Table 1). On average, fruit enzyme activity was very similar in INO and N-INO plants. The only differences between the treatments that are worth emphasizing concerned GR and CAT activity, where nitrogen fertilization was not used. In control treatment GR and CAT activity increased by 12% and 14% in INO plants, respectively. In turn, the APX activity increased by 23% in N-INO plants fertilized with a dose of 100 kg N ha^−1^ (N3). Similar remark can be paid to ascorbate and phenols, the increase in the concentration of these compounds in N0 (INO plants) was by 12% and 7%, respectively. This may signal that the N0 and N3 are less optimal regarding soil environment compared to the other treatments and the simultaneous use of biofertilizer with mineral fertilizer in these combinations activates certain defense systems. Mycorrhizal plants have higher antioxidant enzymes activity compared to non-mycorrhizal plants in several crops [51,52,53,59]. However, these various plants were treated with AMF/PGPR with conjunction with different level of water limitation (drought stress). Plants counteracted to water deficit-induced oxidative stress by upregulating ROS-scavenging antioxidant machinery. Another problem is that the tested bio-fertilizers differ from microorganism composition, which, in combination with naturally occurring microorganisms, may give an effect strongly related to the plant and the layout of applied factors which cannot be directly compared. Pereira et al. [60] tested several plant tissue components with respect to bio-fertilizers and biostimulants reported inconclusive results of the impact on spinach nutritional value and chemical composition suggesting further research in this area. Regarding non-enzymatic antioxidant, in this study neither the different N doses nor the applied inoculum influenced the ascorbate content in the apple peel. The total ascorbate content of ascorbate ranged from 3.87 (N3) to 4.22 g kg^−1^ DW (N4) (Table 2). Total phenolic concentration was also slightly affected by N fertilization. The highest content of phenolic compounds was recorded in the control treatment. However, the decrease in phenolics content was not strictly dose dependent. Significantly lower concentration of total phenol concentrations was recorded at a dose of 50 kg N ha^−1^, which was used in the herbicide fallow only (N1). Other examined treatments did not differ significantly.

Similar results were found in five-year studies [57], where the use of soil and foliar application of nitrogen and potassium fertilizers did not have a strong effect on the concentration of ascorbic acid and phenolic compounds in the apple fruits. It was found that under high N nutrition in apple trees, shoot growth was enhanced and the activity of phenylalanine ammonia lyase, a key enzyme involved in flavonoid biosynthesis, seemed to be downregulated, resulting in a generally decreased flavonoid accumulation [20,61]. In turn, the microorganism can enhance some enzymes involved in phenols and flavonoids metabolism [62]. However, looking at the results of various studies, this process could be modified by accompanying factors.

According to other studies N fertilization caused a decrease in the content of antioxidants such as ascorbate, phenolic compounds and further total antioxidant capacity [4,8,27,45]. Ochoa-Velasco et al., [27] found highly negative correlation between N fertilization and the concentration of total phenolics, total flavonoids, ascorbate and total antioxidant activity, but when N mineral fertilization was accompanied by the use of biofertilizer, the negative correlation was, with an exception of total flavonoids, lowered. Such an effect in the context of N management is promising since low N fertilization can reduce yield formation, which in turn might create a “conflict of interests” between fruit quantity and quality for producers. Moreover, it seems necessary to test the fertilization range together with their impact on pro-healthy compound metabolism to find an optimal quantity and quality yield point with respect to particular species/cultivar [4]. On the other hand the response to N fertilization may also be positive or neutral [49,57,63].

A review of studies regarding effects of the co-inoculation with AMF and PGPR on fruit yield and quality was recently summarized by Jiménez-Gómez et al. [64] and Noceto et al. [2]. The effect on the concentration of ascorbate or phenolic compound was very diversified, either it had no effect or an increase in the concentration of these compounds was noted, less often a decrease. Unfortunately, the number of studied species was limited.

In our previous study on inoculated apple tree in organic system production [34], the antioxidant metabolism was highly dependent on tissue type, growing season and cultivar. The greatest effect on enzymatic and non-enzymatic antioxidants following application of the inoculum was found in roots, then leaves, but it was almost negligible in fruit. Currently presented research results seem to confirm the earlier findings that the effect of microbial inoculum as a tool for enhancing health-promoting properties in the fruit of perennial plants is weaker than that described for vegetables where different plant organs are edible [2,64]. It is likely that in case of trees, the microorganisms based effect is focused rather on the rhizosphere and by this only indirectly influence fruit quality. Therefore, an effect on fruit pro-healthy compounds may be insignificant [34]. Bai et al. [6] emphasized, the change of fertilization of some mineral nutrients will affect the absorption and utilization of other elements and the plants would communicate with rhizosphere microorganisms to improve the absorption and utilization of nutrients; thus, more attention should be paid to the molecular mechanisms of the interactions between different nutrients or plants and microbes and take advantage of them [6]. Another shortcoming is the number of considered perennial plant species regarding microorganism and food quality is limited [2,7].

As phenols are phytochemicals of various structure and activity [20], the determination of their total concentration may not reflect the N-effect and/or inoculation effects on individual compounds. Thus, selected phenolic compounds were determined using HPLC technique. Rutin was detected to have the highest concentration, while caffeic acid had the lowest concentration (Table 3).

The final effect of N fertilization on the concentration of individual phenols depended on the compound, dose and the way of N application. The highest single N dose (N3) caused a significant decrease in (+)-catechins and phloridzin in comparison with the control (Table 3). The range of the decrease ranged from 9 (N1) to 28% (N3) and from 5 (N1) to 25% (N3) for (+)-catechins and phloridzin, respectively.

In case of chlorogenic acid, its concentration decreased under the influence of N fertilization; the decrease range was from 9 (N1) to 20% (N2 and N3) compared to the control, but, at N4, its concentration unexpectedly increased by approx. 8%. In the case of caffeic acid, a 20% and 40% increase in its concentration was recorded, respectively, in the control combination (N0) and at a dose of 50 kg N ha^−1^ applied to the entire plot area (N2) compared to other combinations. The rutin concentration was at the same level, regardless of the N dose and the method of its application (Table 3).

The effect of inoculation on the concentration of individual phenolic compounds has not been proven. However, one observation needs attention. Namely, attention should be paid to changes in the rutin concentration in relation to the use or not bacterial-fungal inoculum and N fertilization. In the range of 24–27%, an increase in rutin concentration was noted in all nitrogen N-INO treatments. In contrast, under control conditions (without N fertilization), rutin increase, on similar to above level, in INO treatment was noted. This compound-dependent pattern of inoculation effect shows that for phenolic compounds, the biofertilizer impact assessment will be difficult and the determination of the total concentration may not be an accurate parameter in this evaluation. We were unable to find more detailed studies regarding the metabolism of individual phenolic compounds and biofertilizers.

## 4. Conclusions

This study revealed that out of the three factors tested, the growing season effect was most pronounced, while the N dose or applied bacterial-fungal inoculum effects were much weaker. Enzymatic antioxidants were more sensitive to changing weather conditions than non-enzymatic ones. Variation in the APX and GR activity was similar regarding the analyzed growing seasons what could be related to high stability of the global ascorbate concentration. The total concentration of phenolic compounds was similar in the first two of the analyzed seasons and then significantly decreased. Since the average temperatures in all seasons were higher (on average for approx. 1.5 °C) than the long-term average, the key factor influencing the antioxidant metabolism seems to be the sum and distribution of precipitation during the growing period. Nitrogen fertilization reduced the fruit’s global phenolic accumulation compared to the control, but the N-effect varied depending on individual phenolic compounds, N dose and N application method. There was a trend of decreasing in (+)-catechin, (−)-epicatechin, chlorogenic acid and phloridzin under the influence of mineral N nutrition; rutin was not affected. However, it should be added that not all phenolic compounds have been determined, especially in the flavanol subgroup, the analysis should be extended to oligomeric flavanols (procyanidins) which have a high share in apple peel phenolics. N fertilizer or biofertilizer effect on this phenolic group should be further explored. Another recommendation would be to express the total concentration of phenolics not in gallic acid because its amount in apples is low and to use a more representative compound, e.g., (−)-epicatechin, which may also influence the tested relationships. Furthermore, on the basis of the results obtained, it is difficult to assess the relationship between N treatments and activity of antioxidant enzymes. In turn, there was a certain tendency to increase antioxidant properties in the control group (without mineral N fertilization) but with the application of bio-fertilizer what may seem promising for future research in this scope. None of the tested factors influenced the ascorbate concentration. In conclusion, it can be stated that the influence of effective microorganisms on the fruit quality is not as obvious as their influence on the plant nutritional status, especially in perennial plants case. This practice still needs to be better investigated with respect to plant species, soil characteristics, crop management and with a more detailed insight into tissue chemical composition or biochemical processes. All studies on fertilization/bio-fertilization and antioxidant metabolism show that these issues are not easy to solve, especially in the field condition. It is because of both the high antioxidant dynamics in plant tissues and the dynamics of the soil environment in relation to essential nutrient circulation and in turn these processes are under influence of microorganism consortium and weather condition.

## Figures and Tables

**Table 1:** Growing season effect on apple peel component tested.

	Antioxidative Enzyme Activity (nkat g^−1^ DW)			Antioxidant Content (g kg^−1^ DW)	
Year	Glutathione Reductase	Ascorbate Proxidase	Catalase	Ascorbate ^1^	Phenolics ^2^
2012	23.8 ^b^	483 ^b^	36.1 ^c^	4.17 ^a^	16.2 ^b^
2013	18.3 ^a^	190 ^a^	27.8 ^b^	3.82 ^a^	16.1 ^b^
2014	30.4 ^c^	549 ^c^	19.6 ^a^	4.16 ^a^	13.7 ^a^

^1^ total ascorbate content, ^2^ total phenolic content expressed as equivalent of gallic acid. DW—dry weight. Means followed by the same superscript letter in column do not differ significantly; Tukey test (*p* < 0.05); data are means through all fertilization treatments in a given year.

**Table 2:** Apple peel antioxidant status: N-fertilization and bacterial–fungal inoculation effects.

N-Treatment ^c^						
	N0	N1	N2	N3	N4	
			GR	nkat g^−1^ DW		Av^N-INOvsINO^
N-INO ^a^	21.7 ± 5.9	22.9 ± 4.4	23.5 ± 4.6	27.2 ± 10.0	24.4 ± 7.5	15.5 ^a^
INO ^b^	24.4 ± 7.0	21.0 ± 5.8	24.4 ± 4.0	26.6 ± 9.5	25.4 ± 12.2	15.2 ^a^
Av^N-treatment^	23.1 ^a^	22.0 ^a^	24.0 ^ab^	27.0 ^b^	24.9 ^ab^	
			CAT	nkat g^−1^ DW		
N-INO	25.6 ± 10.6	29.7 ± 11.9	31.0 ± 10.4	25.6 ± 6.7	27.4 ± 10.8	27.9 ^a^
INO	29.3 ± 9.5	25.8 ± 8.3	30.4 ± 8.5	26.4 ± 1.1	27.2 ± 11.5	27.8 ^a^
Av^N-treatment^	27.5 ^ab^	27.8 ^ab^	30.7 ^b^	26.0 ^a^	27.3 ^ab^	
			APX	nkat g^−1^ DW		
N-INO	357 ± 143	427 ± 213	360 ± 117	504 ± 272	393 ± 188	408 ^a^
INO	346 ± 167	391 ± 186	391 ± 194	409 ± 259	492 ± 302	406 ^a^
Av^N-treatment^	352 ^a^	409 ^abc^	376 ^ab^	456 ^c^	443 ^bc^	
			ASC	g kg^−1^ DW		
N-INO	3.92 ± 0.75	4.24 ± 1.23	4.22 ± 0.38	4.10 ± 0.53	4.03 ± 0.40	4.10 ^a^
INO	4.38 ± 0.85	3.64 ± 0.62	3.91 ± 0.67	3.64 ± 0.65	4.42 ± 0.67	4.00 ^a^
Av^N-treatment^	4.15 ^a^	3.94 ^a^	4.07 ^a^	3.87 ^a^	4.22 ^a^	
			TPC	g kg^−1^ DW		
N-INO	15.5 ± 1.9	14.7 ± 2.1	15.9 ± 2.1	15.7 ± 2.7	15.5 ± 2.6	15.5 ^a^
INO	16.6 ± 2.3	14.3 ± 1.6	14.8 ± 1.8	15.2 ± 1.9	14.9 ± 2.7	15.2 ^a^
Av^N-treatment^	16.0 ^b^	14.5 ^a^	15.4 ^ab^	15.5 ^ab^	15.2 ^ab^	

^a^ N-INO: non-inoculated plants; ^b^ INO: inoculated plants; Means followed by the same superscript letter in column (N-INO vs. INO plants) or N-fertilization (in lines, Av^N-treatment^) do not differ significantly (LSD test); DW—dry weight. ^c^ N0: control, without N fertilization; N1: 50 kg N·ha^−1^ applied for the herbicide fallow N2: 50 kg N·ha^−1^ the entire plot surface was fertilized; N3: 100 kg N·ha^−1^ the entire plot surface was fertilized, N4: in total 100 kg N·ha^−1^ was applied, but divided into two doses: the first dose of 50 kg N·ha^−1^ was applied to the entire surface of the plot in early spring and the second one of 50 kg N·ha^−1^ at the end of May. The presented data are averages through applied fertilization treatments and examined growing seasons (means ± SD, n = 9).

**Table 3:** Individual phenolic compound concentration depending on N-dose and its way of application in inoculated and non-inoculated plants (g kg^−1^ DW).

	N-Treatment ^c^					
	N0	N1	N2	N3	N4	
			(+)-Catechin			Av^N-INOvsINO^
N-INO ^a^	0.23 ± 0.02	0.22 ± 0.01	0.23 ± 0.04	0.19 ± 0.03	0.20 ± 0.02	0.21 ^a^
INO ^b^	0.24 ± 0.04	0.20 ± 0.00	0.15 ± 0.06	0.17 ± 0.02	0.20 ± 0.08	0.19 ^a^
Av^N-treatment^	0.23 ^b^	0.21 ^b^	0.19 ^ab^	0.18 ^a^	0.20 ^ab^	
Chlorogenic acid						
N-INO	0.12 ± 0.02	0.10 ± 0.02	0.11 ± 0.01	0.11 ± 0.00	0.13 ± 0.02	0.11 ^a^
INO	0.13 ± 0.02	0.10 ± 0.01	0.10 ± 0.03	0.10 ± 0.01	0.13 ± 0.01	0.11 ^a^
Av^N-treatment^	0.12 ^bc^	0.10 ^a^	0.11 ^ab^	0.11 ^ab^	0.13 ^c^	
(−)-Epicatechin						
N-INO	2.79 ± 0.25	2.57 ± 0.32	2.61 ± 0.07	2.77 ± 0.13	2.77 ± 0.49	2.70 ^a^
INO	3.25 ± 0.50	2.73 ± 0.23	2.46 ± 0.36	2.53 ± 0.26	2.41 ± 0.66	2.67 ^a^
Av^N-treatment^	3.02 ^b^	2.65 ^ab^	2.53 ^a^	2.65 ^ab^	2.59 ^ab^	
Caffeic acid						
N-INO	0.06 ± 0.00	0.04 ± 0.01	0.06 ± 0.00	0.05 ± 0.01	0.06 ± 0.00	0.53 ^a^
INO	0.07 ± 0.01	0.06 ± 0.01	0.07 ± 0.03	0.05 ± 0.01	0.05 ± 0.02	0.58 ^a^
Av^N-treatment^	0.06 ^ab^	0.05 ^a^	0.07 ^b^	0.05 ^a^	0.05 ^a^	
			Rutin			
N-INO	4.64 ± 0.77	5.23 ± 2.81	5.17 ± 0.58	5.18 ± 0.52	5.18 ± 1.59	5.08 ^a^
INO	5.73 ± 0.58	4.17 ± 0.83	4.14 ± 1.08	4.10 ± 1.09	4.05 ± 0.26	4.64 ^a^
Av^N-treatment^	5.19 ^a^	4.70 ^a^	4.65 ^a^	4.64 ^a^	5.12 ^a^	
			Phloridzin			
N-INO	0.37 ± 0.03	0.39 ± 0.08	0.34 ± 0.05	0.32 ± 0.05	0.33 ± 0.03	0.35 ^a^
INO	0.42 ± 0.07	0.38 ± 0.06	0.35 ± 0.04	0.33 ± 0.03	0.38 ± 0.05	0.37 ^a^
Av^N-treatment^	0.40 ^b^	0.38 ^ab^	0.34 ^ab^	0.32 ^a^	0.36 ^ab^	

^a^ N-INO: non-inoculated plants; ^b^ INO: inoculated plants; Means followed by the same super-script letter in column (N-INO vs. INO plants) or N-fertilization (in lines, Av^N-treatment^) do not differ significantly (LSD test); DW—dry weight. ^c^ N0: control, without N fertilization; N1: 50 kg N·ha^−1^ applied to for the herbicide fallow; N2: 50 kg N·ha^−1^, the entire plot surface was fertilized; N3: 100 kg N·ha^−1^, the entire plot surface was fertilized, N4: in total 100 kg N·ha^−1^ was applied, but divided into two doses: the first dose (50 kg N·ha^−1^) was applied to the entire surface of the plot in early spring and the second one (50 kg N·ha^−1^) at the end of May.

## Data Availability

The data are contained within the article.

## References

[1] Poiroux-Gonord F., Bidel L.P.R., Fanciullino A.-L., Gautier H., Lauri-Lopez F., Urban L. (2010). Health benefits of vitamins and secondary metabolites of fruits and vegetables and prospects to increase their concentrations by agronomic approaches. J. Agric. Food Chem..

[2] Noceto P.A., Bettenfeld P., Boussageon R., Hériché M., Sportes A., van Tuinen D., Courty P.E., Wipf D. (2021). Arbuscular mycorrhizal fungi, a key symbiosis in the development of quality traits in crop production, alone or combined with plant growth-promoting bacteria. Mycorrhiza.

[3] Srednicka-Tober D., Baranski M., Kazimierczak R., Ponder A., Kopczynska K., Hallmann E. (2020). Selected antioxidants in organic vs. conventionally grown apple fruits. Appl. Sci..

[4] Stefaniak J., Przybył J.L., Latocha P., Łata B. (2020). Bioactive compounds, total antioxidant capacity and yield of kiwiberry fruit under different nitrogen regimes in field conditions. J. Sci. Food Agric..

[5] Milošević T., Milošević N., Mladenović J. (2019). Tree vigor, yield, fruit quality, and antioxidant capacity of apple (Malus × domestica borkh.) influenced by different fertilization regimes: Preliminary results. Turk. J. Agric. For..

[6] Bai Q., Shen Y., Huang Y. (2021). Advances in Mineral Nutrition Transport and Signal Transduction in Rosaceae Fruit Quality and Postharvest Storage. Front. Plant Sci..

[7] Tang H., Hassan M.U., Feng L., Nawaz M., Shah A.N., Qari S.H., Liu Y., Miao J. (2022). The Critical Role of Arbuscular Mycorrhizal Fungi to Improve Drought Tolerance and Nitrogen Use Efficiency in Crops. Front. Plant Sci..

[8] Barreto C.F., Navroski R., Ferreira L.V., Benati J.A., Malgarim M.B., Antunes L.E.C. (2020). Nitrogen fertilization associated with cold storage and its impacts on the maintenance of peach quality. Biosci. J..

[9] Jezek M., Zörb C., Merkt N., Geilfus C.M. (2018). Anthocyanin Management in Fruits by Fertilization. J. Agric. Food Chem..

[10] Tavanti T.R., de Melo A.A.R., Moreira L.D.K., Sanchez D.E.J., Silva R.D.S., da Silva R.M., Reis A.R. (2021). Micronutrient fertilization enhances ROS scavenging system for alleviation of abiotic stresses in plants. Plant Physiol. Biochem. PPB.

[11] Foyer C.H., Noctor G. (2011). Ascorbate and glutathione: The heart of the redox hub. Plant Physiol..

[12] Foyer C.H. (2020). How plant cells sense the outside world through hydrogen peroxide. Nature.

[13] An Y., Sun H., Zhang W., Sun Y., Li S., Yu Z., Yang R., Hu T., Yang P. (2022). Distinct rhizosphere soil responses to nitrogen in relation to microbial biomass and community composition at initial flowering stages of alfalfa cultivars. Front. Plant Sci..

[14] Erisman J.W., Leach A., Bleeker A., Atwell B., Cattaneo L., Galloway J. (2018). An integrated approach to a nitrogen use efficiency (NUE) indicator for the food production-consumption chain. Sustainability.

[15] Jain G. (2019). National Seminar “Role of Biological Sciences in Organic Farming” Role of micronutrients in potato cultivation. J. Pharmacogn. Phytochem..

[16] Ye X., Abe S., Zhang S. (2020). Estimation and mapping of nitrogen content in apple trees at leaf and canopy levels using hyperspectral imaging. Precis. Agric..

[17] Adesemoye A.O., Torbert H.A., Kloepper J.W. (2009). Plant growth-promoting rhizobacteria allow reduced application rates of chemical fertilizers. Microb. Ecol..

[18] Ganugi P., Fiorini A., Rocchetti G., Bonini P., Tabaglio V., Lucini L. (2022). A response surface methodology approach to improve nitrogen use efficiency in maize by an optimal mycorrhiza-to-Bacillus co-inoculation rate. Front. Plant Sci..

[19] Chaudhary P., Singh S., Chaudhary A., Sharma A., Kumar G. (2022). Overview of biofertilizers in crop production and stress management for sustainable agriculture. Front. Plant Sci..

[20] Shah A., Smith D.L. (2020). Flavonoids in agriculture: Chemistry and roles in, biotic and abiotic stress responses, and microbial associations. Agronomy.

[21] Bokszczanin K.Ł., Wrona D., Przybyłko S. (2021). The Effect of Microbial Inoculation under Various Nitrogen Regimes on the Uptake of Nutrients by Apple Trees. Agronomy.

[22] Mitra D., Djebaili R., Pellegrini M., Mahakur B., Sarker A., Chaudhary P., Khoshru B., Del Gallo M., Kitouni M., Barik D.P. (2021). Arbuscular mycorrhizal symbiosis: Plant growth improvement and induction of resistance under stressful conditions. J. Plant Nutr..

[23] Egamberdieva D., Wirth S.J., Alqarawi A.A., Abd Allah E.F., Hashem A. (2017). Phytohormones and Beneficial Microbes: Essential Components for Plants to Balance Stress and Fitness. Front. Microbiol..

[24] Zydlik Z., Zydlik P., Wieczorek R. (2021). The Effects of Bioinoculants Based on Mycorrhizal and Trichoderma spp. Fungi in an Apple Tree Nursery under Replantation Conditions. Agronomy.

[25] Lambers H., Raven J.A., Shaver G.R., Smith S.E. (2008). Plant nutrient-acquisition strategies change with soil age. Trends Ecol. Evol..

[26] Zhu L., Huang J., Lu X., Zhou C. (2022). Development of plant systemic resistance by beneficial rhizobacteria: Recognition, initiation, elicitation and regulation. Front. Plant Sci..

[27] Ochoa-Velasco C.E., Valadez-Blanco R., Salas-Coronado R., Sustaita-Rivera F., Hernández-Carlos B., García-Ortega S., Santos-Sánchez N.F. (2016). Effect of nitrogen fertilization and Bacillus licheniformis biofertilizer addition on the antioxidants compounds and antioxidant activity of greenhouse cultivated tomato fruits (*Solanum lycopersicum* L. var. Sheva). Sci. Hortic..

[28] Kumar S., Sharma A., Sharma V.K., Rosin K.G., Kumar D. (2018). Enhancement of apple (*Malus domestica*) productivity and soil health through organic fertilization and bio-inoculants under north-western Himalayan region of India. Indian J. Agric. Sci..

[29] Kuzin A., Solovchenko A., Stepantsova L., Pugachev G. (2020). Soil fertility management in apple orchard with microbial biofertilizers. E3S Web Conf..

[30] Treder W., Klamkowski K., Wójcik K., Tryngiel-Gać A., Sas-Paszt L., Mika A., Kowalczyk W. (2022). Apple leaf macro- and micronutrient content as affected by soil treatments with fertilizers and microorganisms. Sci. Hortic..

[31] Khan M.Y., Haque M.M., Molla A.H., Rahman M.M., Alam M.Z. (2017). Antioxidant compounds and minerals in tomatoes by Trichoderma-enriched biofertilizer and their relationship with the soil environments. J. Integr. Agric..

[32] Zahedyan A., Aboutalebi Jahromi A., Zakerin A., Abdossi V., Mohammadi Torkashvand A. (2022). Nitroxin bio-fertilizer improves growth parameters, physiological and biochemical attributes of cantaloupe (*Cucumis melo* L.) under water stress conditions. J. Saudi Soc. Agric. Sci..

[33] Mohamed M.H.M., Ali M., Eid R.S.M., El-Desouky H.S., Petropoulos S.A., Sami R., Al-Mushhin A.A.M., Ismail K.A., Zewail R.M.Y. (2021). Phosphorus and biofertilizer application effects on growth parameters, yield and chemical constituents of broccoli. Agronomy.

[34] Łata B., Łaźny R., Przybyłko S., Wrona D. (2021). Malus antioxidant metabolism following bacterial–fungal inoculation in organic farming: From root to fruit. Appl. Sci..

[35] Sun T., Zhang J., Zhang Q., Li X., Li M., Yang Y., Zhou J., Wei Q., Zhou B. (2021). Integrative physiological, transcriptome, and metabolome analysis reveals the effects of nitrogen sufficiency and deficiency conditions in apple leaves and roots. Environ. Exp. Bot..

[36] Wu Q.-S., Xia R.-X., Zou Y.-N. (2006). Reactive oxygen metabolism in mycorrhizal and non-mycorrhizal citrus (*Poncirus trifoliata*) seedlings subjected to water stress. J. Plant Physiol..

[37] Garza-Alonso C.A., Nino-Medina G., Guttierez-Diez A., Garcia-Lopez J.I., Vazquez-Alvarado R.E., Lopez-Jimenez A., Olivares-Saenz E. (2020). Physicochemical characteristics, minerals, phenolic compounds, and antioxidant capacity in fig tree fruits with macronutrient deficiencies. Not. Bot. Horti Agrobot. Cluj-Napoca.

[38] You J., Chan Z. (2015). ROS Regulation During Abiotic Stress Responses in Crop Plants. Front. Plant Sci..

[39] Yuri J.A., Neira A., Fuentes M., Sáez B., Razmilic I. (2022). Have the Flowers, Fruitlets, Ripe Fruit and Leaves of Apples Cultivars Similar Compositions of Phenolic and Antioxidant Capacity?. Erwerbs-Obstbau.

[40] Łata B., Przeradzka M., Bínkowska M. (2005). Great differences in antioxidant properties exist between 56 apple cultivars and vegetation seasons. J. Agric. Food Chem..

[41] Łata B. (2007). Relationship between apple peel and the whole fruit antioxidant content: Year and cultivar variation. J. Agric. Food Chem..

[42] Musacchi S., Serra S. (2018). Apple fruit quality: Overview on pre-harvest factors. Sci. Hortic..

[43] Li P., Ma F., Cheng L. (2013). Primary and secondary metabolism in the sun-exposed peel and the shaded peel of apple fruit. Physiol. Plant..

[44] Marranzano M., Rosa R.L., Malaguarnera M., Palmeri R., Tessitori M., Barbera A.C. (2018). Polyphenols: Plant Sources and Food Industry Applications. Curr. Pharm. Des..

[45] Torres N., Hilbert G., Antolín M.C., Goicoechea N. (2019). Aminoacids and Flavonoids Profiling in Tempranillo Berries Can Be Modulated by the Arbuscular Mycorrhizal Fungi. Plants.

[46] Kumar S., Kundu M., Das A., Rakshit R., Siddiqui M.W., Rani R. (2019). Substitution of mineral fertilizers with biofertilizer: An alternate to improve the growth, yield and functional biochemical properties of strawberry (*Fragaria × ananassa* Duch.) cv. Camarosa. J. Plant Nutr..

[47] Medina M.B. (2011). Determination of the total phenolics in juices and superfruits by a novel chemical method. J. Funct. Foods.

[48] Łata B., Trampczynska A., Paczesna J. (2009). Cultivar variation in apple peel and whole fruit phenolic composition. Sci. Hortic..

[49] Łata B. (2014). Variability in enzymatic and non-enzymatic antioxidants in red and green-leafy kale in relation to soil type and N-level. Sci. Hortic..

[50] Hanaka A., Ozimek E., Reszczyńska E., Jaroszuk-ściseł J., Stolarz M. (2021). Plant tolerance to drought stress in the presence of supporting bacteria and fungi: An efficient strategy in horticulture. Horticulturae.

[51] Mandavikia H., Rezaei-Chiyaneh E., Rahimi A., Mohammadkhani N. (2019). Effects of Fertilizer Treatments on Antioxidant Activities and Physiological Traits of Basil (*Ocimum basilicum* L.) under Water Limitation Conditions. J. Med. Plants By-Products-JMPB.

[52] Anli M., Baslam M., Tahiri A., Raklami A., Symanczik S., Boutasknit A., Ait-El-Mokhtar M., Ben-Laouane R., Toubali S., Ait Rahou Y. (2020). Biofertilizers as Strategies to Improve Photosynthetic Apparatus, Growth, and Drought Stress Tolerance in the Date Palm. Front. Plant Sci..

[53] Sheteiwy M.S., Ali D.F.I., Xiong Y.C., Brestic M., Skalicky M., Hamoud Y.A., Ulhassan Z., Shaghaleh H., AbdElgawad H., Farooq M. (2021). Physiological and biochemical responses of soybean plants inoculated with Arbuscular mycorrhizal fungi and Bradyrhizobium under drought stress. BMC Plant Biol..

[54] Mignard P., Beguería S., Reig G., Font i Forcada C., Moreno M.A. (2021). Genetic origin and climate determine fruit quality and antioxidant traits on apple (*Malus x domestica* Borkh). Sci. Hortic..

[55] Li M., Ma F., Shang P., Zhang M., Hou C., Liang D. (2009). Influence of light on ascorbate formation and metabolism in apple fruits. Planta.

[56] Bui T.T.A., Wright S.A.I., Falk A.B., Vanwalleghem T., Van Hemelrijck W., Hertog M.L., Keulemans J., Davey M.W. (2019). Botrytis cinerea differentially induces postharvest antioxidant responses in ‘Braeburn’ and ‘Golden Delicious’ apple fruit. J. Sci. Food Agric..

[57] Vetrova O., Makarkina M., Larisa L. (2022). The effect of mineral nutrition on the biochemical composition of Venyaminovskoe apple cultivar fruits. BIO Web Conf..

[58] Carranca C., Brunetto G., Tagliavini M. (2018). Nitrogen Nutrition of Fruit Trees to Reconcile Productivity and Environmental Concerns. Plants.

[59] Najafi S., Nasi H.N., Tuncturk R., Tuncturk M., Sayyed R.Z., Amirnia R. (2021). Biofertilizer application enhances drought stress tolerance and alters the antioxidant enzymes in medicinal pumpkin (*Cucurbita pepo* convar. pepo var. Styriaca). Horticulturae.

[60] Pereira C., Dias M.I., Petropoulos S.A., Plexida S., Chrysargyris A., Tzortzakis N., Calhelha R.C., Ivanov M., Stojković D., Soković M. (2019). The effects of biostimulants, biofertilizers and water-stress on nutritional value and chemical composition of two spinach genotypes (*Spinacia oleracea* L.). Molecules.

[61] Strissel T., Halbwirth H., Hoyer U., Zistler C., Stich K., Treutter D. (2005). Growth-promoting nitrogen nutrition affects flavonoid biosynthesis in young apple (*Malus domestica* Borkh.) leaves. Plant Biol..

[62] Narendra Babu A., Jogaiah S., Ito S.-I., Kestur Nagaraj A., Tran L.-S.P. (2015). Improvement of growth, fruit weight and early blight disease protection of tomato plants by rhizosphere bacteria is correlated with their beneficial traits and induced biosynthesis of antioxidant peroxidase and polyphenol oxidase. Plant Sci..

[63] Peñas E., Zielińska D., Gulewicz P., Zieliński H., Frias J. (2018). Vitamin C, Phenolic Compounds and Antioxidant Capacity of Broccoli Florets Grown under Different Nitrogen Treatments Combined with Selenium. Pol. J. Food Nutr. Sci..

[64] Jiménez-Gómez A., Celador-Lera L., Fradejas-Bayón M., Rivas R. (2017). Plant probiotic bacteria enhance the quality of fruit and horticultural crops. AIMS Microbiol..

